# Deciphering the Two-Step Hydride Mechanism of Monoamine Oxidase Flavoenzymes

**DOI:** 10.1021/acsomega.4c06575

**Published:** 2024-10-10

**Authors:** Martina Rajić, Alja Prah, Jernej Stare

**Affiliations:** Theory Department, Laboratory for Computational Biochemistry and Drug Design, National Institute of Chemistry, Hajdrihova 19, Ljubljana SI-1000, Slovenia

## Abstract

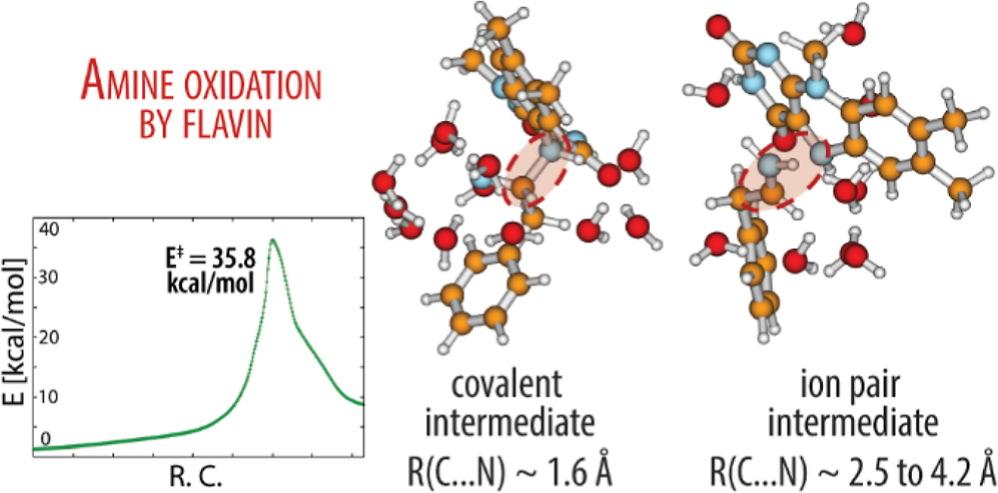

The complete two-step hydride transfer mechanism of amine oxidation involved in the metabolism of monoamine neurotransmitters was scrutinized by DFT calculations. In living organisms, this process is catalyzed by monoamine oxidase enzymes. Herein, we focus on some intriguing aspects of the reaction that may have been previously noticed but have not been clarified to date. The first step of the reaction includes the C–H bond cleavage on the methylene group vicinal to the amino group of the monoamine substrate and the subsequent transfer of hydrogen to the N5 atom of the flavin prosthetic group of the enzyme. We confirmed the nature of this step to be hydride transfer by evaluation of the pertinent HOMO–LUMO gap together with analysis of orbital contours alongside the intrinsic reaction coordinate profile. Next, we investigated the rather peculiar intermediate adduct that may form between the amine substrate and the flavin molecule, featuring an unusually long C–N bond of ∼1.62 Å. Although this bond is quite stable in the gas phase, the presence of just a few explicit water molecules facilitates its dissociation almost without energy input so that the amine-flavin intermediate can form an ionic pair instead. We attribute the existence of the unusual C–N bond to a fragile balance between opposing electronic structure effects, as evaluated by the natural bond orbital analysis. In line with this, the intermediate in the solution or in the enzyme active site can exist in two energetically almost equivalent forms, namely, as a covalently bound complex or as an ion pair, as suggested by previous studies. Finally, we characterized the transformation of the intermediate to the fully reduced flavin and imine products via proton transfer from the amino group to the flavin N1 atom, completing the reductive part of the catalytic cycle. Although we found that explicit solvation substantially boosts the kinetics of this step, the corresponding barrier is significantly lower than that in the hydride transfer step, confirming hydrogen abstraction as the rate-limiting step of amine oxidation and validating the two-step hydride transfer mechanism of monoamine oxidases.

## 1 Introduction

**Figure 1:**
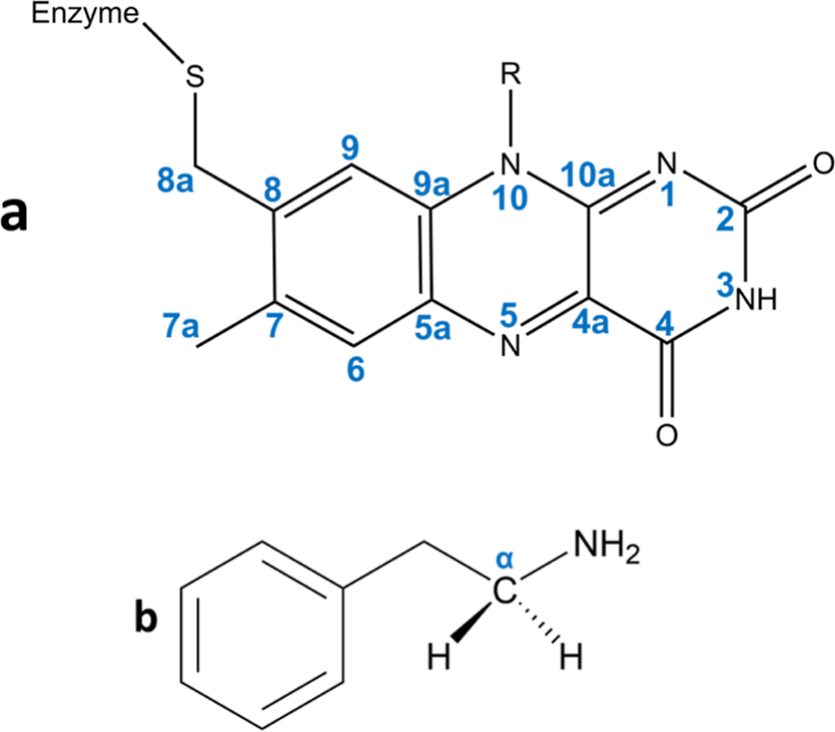
Structure and atom numbering of the flavin moiety of FAD (a) and the herein studied substrate phenylethylamine (PEA) with the reacting carbon located at the vicinal position relative to the amino group (C_α_) indicated (b).

Monoamine oxidases (MAOs) A and B are mitochondrial outer membrane-bound isoenzymes that catalyze the oxidative deamination of a great deal of biogenic amines, including neurotransmitters dopamine, serotonin, and noradrenaline,^1,2^ and also some other biogenic substrates such as phenylethylamine (endogenic neuromodulator)^3,4^ (PEA) and histamine.^5−7^ In addition, they can decompose nonbiogenic substrates such as benzylamine^8^ and various phenylethylamine derivatives, which are used as research substrates of MAOs.^9^ MAO-A and MAO-B exhibit about 70% sequence identity and use the same flavin adenine dinucleotide (FAD) as a cofactor. Therefore, it is generally assumed that they operate by the same mechanism, although this view was challenged in the past.^10^ A general agreement is that the initial and rate-limiting step is the stereospecific transfer of a hydrogen atom bound to the carbon atom vicinal to the amino group (C_α_) of the substrate to the FAD cofactor which is covalently bound to one of the cysteine residues in sequence (see Figure 1 for atomic labels).

**Scheme 1:**
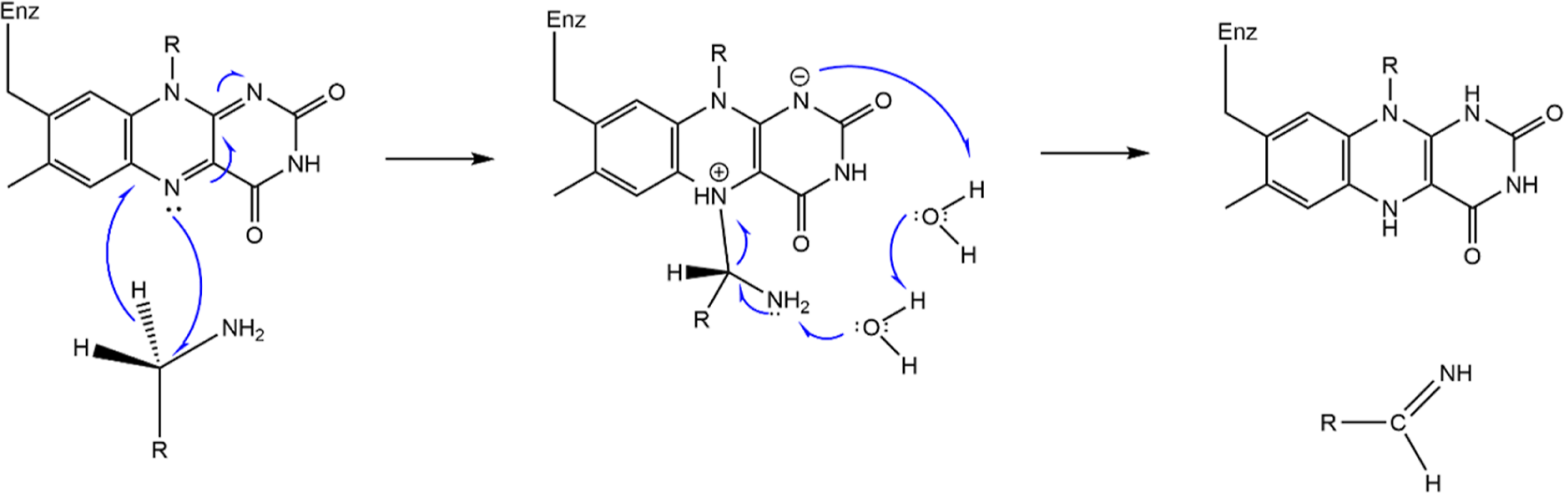
Two-Step Hydride Transfer Mechanism as Proposed by Vianello et al.^19^ Reproduced from [Vianello, R.; Repic, M.; Mavri, J., How are Biogenic Amines Metabolized by Monoamine Oxidases? *Eur. J. Org. Chem.* 2012, *36*, 7057-7065] Copyright 2012, with permission from John Wiley and Sons.

Based on a variety of experimental observations, the following catalytic mechanisms have been proposed for amine oxidation by FAD: polar nucleophilic,^11−13^ radical,^14,15^ direct hydride transfer,^16−18^ and two-step hydride transfer.^19^ These mechanisms have been studied and discussed by several experimental and theoretical investigations,^14−22^ but there has been growing evidence in favor of the latter, namely, the two-step hydride transfer mechanism, for which Vianello et al. demonstrated, by using DFT calculations on cluster models, strong kinetic and thermodynamic preference over all other mechanisms.^19^ The two-step hydride transfer mechanism is presented in Scheme 1.

As implied by the name, the two-step hydride transfer mechanism consists of two distinct steps. In the first step which is rate-limiting (see Scheme 1), the hydrogen atom is abstracted from C_α_ of the substrate and transferred to the flavin N5 atom in the form of a hydride ion (H^–^). This results in a covalently bound intermediate consisting of a semioxidized substrate and semireduced flavin. In the second step, another hydrogen atom is transferred from the substrate to flavin, namely, from the amino group to the N1 atom, but this time as a proton (H^+^) to complete the reaction, yielding an oxidized imine substrate and fully reduced (dihydrogenated) flavin (Scheme 1). Possibly for steric reasons, the second step is assisted by two bridging water molecules providing a proton transfer pathway from the amino group to the N1 atom (Scheme 1).

The two-step hydride transfer mechanism features a barrier of ∼24–26 kcal/mol (depending on substrate), as evaluated with a DFT model mimicking the active site of MAO enzymes by including three explicit tyrosine side chains and four water molecules.^19^ In the gas phase or in an implicit solvent, the barrier appears to be higher, slightly above 30 kcal/mol, as computed occasionally by DFT in the context of a parameterizing simulation of the reaction by the empirical valence bond (EVB) technique. Therefore, Vianello’s study also demonstrates the catalytic role of MAO enzymes in that they facilitate a noticeable lowering of the barrier, thereby boosting the kinetics. Based on Vianello’s findings, several computational studies of MAO reactions assumed the hydride transfer mechanism;^22−26^ in all cases, the very good agreement between the computed and experimental kinetic parameters of wild-type and mutated variants of MAO enzymes gave sound evidence in favor of that mechanism. However, these studies were mainly based on the EVB methodology,^27−29^ which requires the reaction mechanism to be specified in advance. Since these studies included exclusively the presumed rate-limiting step rather than the whole mechanism, the sole validated part, strictly speaking, is that the C_α_–H bond cleavage concerted with hydride transfer from the substrate to flavin likely represents the rate-limiting step, whereas no information about other mechanistic details can be derived.

**Figure 2:**
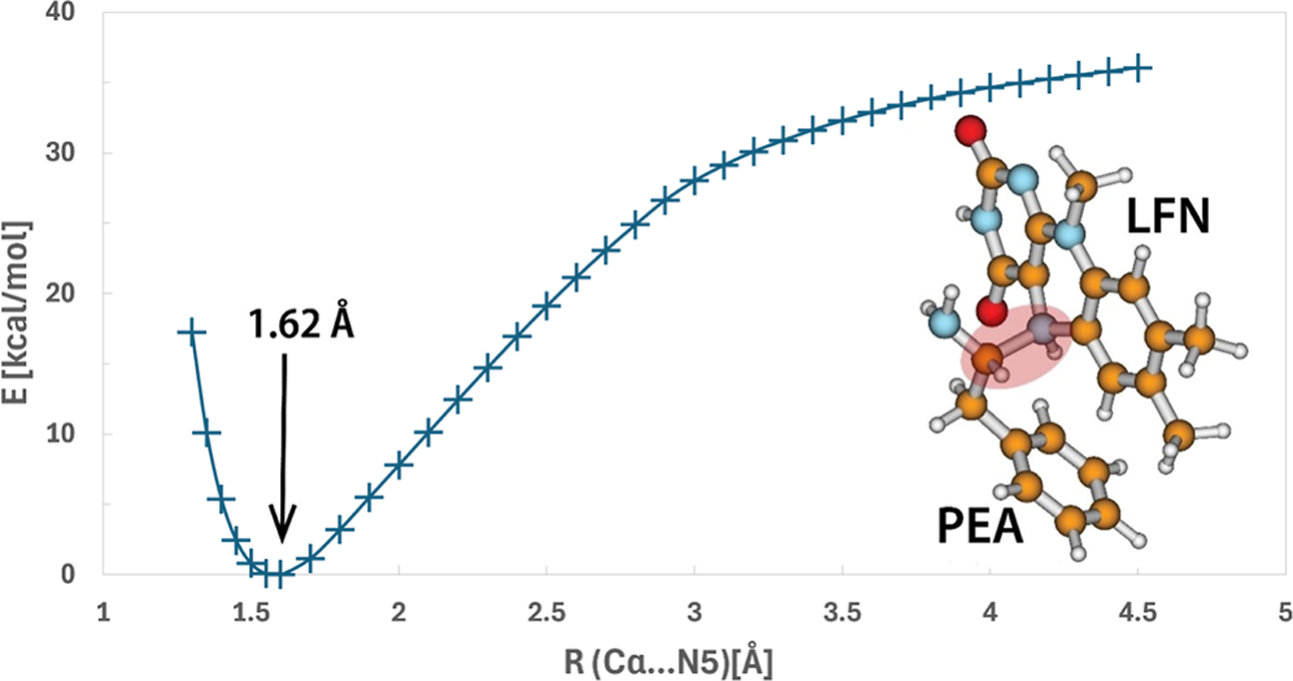
Potential energy function along the C_α_···N5 distance and structure of the covalently bound complex of phenylethylamine (PEA) and lumiflavin (LFN) with the C_α_–N5 bond marked as a red-shaded ellipse. The involved PEA and LFN entities (see Section 2 for an explanation) are indicated as well as the equilibrium C_α_···N5 separation of 1.62 Å.

Despite a thorough computational investigation of various mechanisms using fairly complex models, Vianello’s study leaves certain mechanistic aspects open. Namely, DFT calculations suggest that just after hydrogen (hydride) transfer from the amine substrate to FAD, these molecules form a rather unusual covalent intermediate complex in which the C_α_ atom of the substrate and the N5 atom of flavin are connected by a surprisingly long bond of ∼1.62 Å, which is by ∼0.15 Å longer than a typical (“single”) C–N bond.^30^ Furthermore, the estimated dissociation profile of that bond computed at a preliminary stage of this study (Figure 2) suggests that an energy input of over 30 kcal/mol is required to increase the C_α_···N5 separation to roughly 4 Å (let alone to fully separate the two molecules). This implies that the cleavage of the intermediate complex may exceed in energy the transition state (TS) of the hydrogen transfer in the precedent step; in contrast to previous findings, this could even render the C_α_–N5 bond dissociation the rate-limiting step of the substrate oxidation and, consequently, of the entire catalytic cycle of MAOs. This issue is important for the understanding of the mechanism and requires investigation. Since the involved entities are oppositely charged (positive semioxidized amine substrate and negative semireduced flavin), solvation effects may play a vital role in the energy profile, particularly in the part pertinent to dissociation, and have to be properly undertaken. Moreover, the understanding of the mechanism may be improved by investigating the unusual C_α_–N5 bond, in terms of the underlying electronic structure effects. Also, while the assumed hydride transfer mechanism involves transfer of negative charge from the substrate to flavin, the corresponding changes in charge distribution have not yet been scrutinized in great detail. Finally, the second step of the mechanism, i.e., proton transfer from the substrate to the N1 atom of flavin, also requires attention, in that which factors govern its kinetics. Also, the possibility that both hydride and proton transfer steps occur in a concerted manner, essentially making the mechanism of a single-step type, has not been entirely ruled out. To our knowledge, while the aforementioned peculiar characteristics of the amine oxidation reaction may have been detected, they have not been assessed explicitly.

Interestingly enough, Maršavelski and Vianello found in a related DFT cluster study of histamine (HIS) and *N*-methylhistamine (NMH) oxidation by MAO-B that the intermediate preferably exists in the form of an ion pair with a C_α_···N5 distance of ∼2.6–2.9 Å rather than forming a covalent C_α_–N5 bond.^31^ In part, this opposes findings on the intermediate complex involving dopamine as a substrate in an earlier study.^19^ The authors attribute the notable difference in the structure of the complex between histamine and dopamine to chemical differences between the two compounds. However, in light of the noticeable chemical similarity between monoamine substrates of MAOs (the reacting methylene group and the vicinal amino group are common to practically all endogenous MAO substrates), their findings may also imply that in the enzymatic environment, dissociation of the C_α_–N5 bond is much more feasible than suggested by the observed stability of that bond in the gas phase (Figure 2), possibly requiring low energy inputs for converting the covalently bound intermediate to the ion pair and vice versa. Therefore, we feel that this aspect deserves further attention.

Another important feature devised from the study of Maršavelski and Vianello^31^ is that for the reaction in question, the (polar) environment—whether it is the solvent or the enzyme active site—can have a profound effect on the reaction profile and/or the structure and stability of the intermediate, which alone calls for detailed investigation. Among quantum chemistry protocols available for this purpose, perhaps the most simple and computationally inexpensive is the methodology of implicit solvation, also named self-consistent reaction field (SCRF).^32,33^ This technique accounts for solvation effects by enclosing the system of interest (a solute) in a cavity inside which the free space electrostatics is assumed (with a dielectric constant of 1), whereas outside the cavity, the solvent is represented solely by its corresponding dielectric constant. The solvent and the solute mutually polarize each other, thereby affecting the charge distribution of the solute (and consequently its structure and other properties). In the SCRF representation, the solvent molecules are effectively averaged over all their possible configurations occurring over a long time, but this representation lacks any explicit information on the (instantaneous) molecular and electronic structure of the solvent. The approach is inexpensive and requires a comparable amount of resources to a gas-phase calculation; only the solute molecular structure needs to be specified for an SCRF calculation. For the herein investigated hydride transfer step of amine oxidation by flavin, the free energy of solvation of the TS is slightly higher (more negative) than that of the reactant state, meaning that the barrier in the implicit solvent (typically water) is lower than that in the gas phase, but only slightly (∼4 kcal/mol, see Table 1). Interestingly, implicit solvation barely flattens the C_α_–N5 bond dissociation profile, keeping this part of the reaction energetically demanding (similarly to the gas phase, see Figure 2). As we feel this is unrealistic for the presently studied reaction, the challenge is to use the approach of explicit (rather than implicit) solvation, meaning that individual solvent molecules are present in the model and treated by the same quantum chemistry protocol, which may be more realistic in terms of interactions between the solute and solvent but at the same time exceedingly demanding because of the substantially increased computational cost. Furthermore, because solvent molecules can usually assume a myriad of energetically nearly equivalent conformations, thermal averaging is normally required to account for the conformational flexibility of the solvent. In the present study, we included the modeling of the reaction by applying explicit solvation (limited to 15 surrounding water molecules), and efforts have been made to account for thermal averaging, as explained below.

**Table 1:** Hydride Transfer Reaction Barrier, Energy, and C_α_···N5 Separation in the Product (Intermediate) State Computed for Various Explicitly Solvated Models with Different Numbers of Explicit Water Molecules

model #	no. of water molecules	reaction barrier [kcal/mol]	reaction energy [kcal/mol]	R(C_α_···N5) [Å]
**1**	15	40.4	2.3	1.63
**2**	13	27.0	–7.2	3.73
**3**	12	32.9	–1.2	2.68
**4**	9	33.8	3.4	2.56
**5**	9	38.8	6.5	2.58
**6**	12	35.4	5.8	3.96
**7**	13	28.2	–7.2	3.73
**8**	11	29.4	–4.2	3.54
**9**	12	38.4	6.8	1.59
**10**	14	34.3	1.7	3.64
**11**	13	24.8	–2.1	3.46
**12**	14	35.4	5.7	3.97
**average**	N/A	33.2	0.8	N/A
**SCRF**	0	31.7	2.7	1.65
**GAS**	0	35.8	6.9	1.62

In the present work, we focus on the two-step hydride transfer mechanism using quantum chemical calculations, expanding the already published computational study^19^ by including (i) characterization of the reaction profile of both steps by using the intrinsic reaction coordinate (IRC) method;^34,35^ (ii) analysis of the frontier molecular orbitals and the energy gap between the lowest occupied (HOMO) and highest unoccupied (LUMO) orbital of the reacting molecules to confirm the negative charge transfer during the reaction; (iii) investigation of the unusual C_α_–N5 bond in the intermediate adduct, its dissociation and possible influence of solvation on dissociation; (iv) elucidation of the proton transfer step which completes the reaction, focusing on the factors influencing this step.

## 2 Computational Methods

The reaction mechanism was investigated by DFT calculations using the M06-2X functional developed by Zhao and Truhlar for calculating the barriers of organic reactions,^36^ together with the 6-31+G(d,p) basis set. All calculations were carried out with the *Gaussian16* program package,^37^ and most of them were based on standard optimization procedures. Identity of the computed stationary points was validated by the harmonic frequency check. Selected reaction steps were further characterized by the IRC approach^34,35^ starting from the respective TS structure and carried out in both directions. The Hessian was recomputed every 10 predictor steps to ensure the accuracy of the profiles. In both directions, a sufficiently high maximum number of IRC steps was set to ensure completion of the pathway by reaching the respective minima (reactants and products). In addition, all the minimum-energy structures acquired by IRC were further optimized without restrictions; in case IRC failed to converge to the corresponding minimum, we also applied full optimization starting from the last computed point on the IRC pathway.

The model consisted of the PEA molecule representing a typical substrate of MAO, while the FAD prosthetic group was represented by lumiflavin (LFN), a truncated version of FAD retaining the triple-ring isoalloxazine moiety that is crucial for its functionality (see Figure 2). Apart from PEA, we also used HIS and NMH as substrates in a minor part of the calculations. Solvation effects were included either by applying the implicit SCRF treatment^32,33,38,39^ using water as a solvent or by adding explicit water molecules to the model, as will be explained below. Where needed, the electronic structure of the system was analyzed by means of natural bond orbital (NBO) methodology (v. 3.1),^40−43^ as implemented in *Gaussian*.

For the first of the presumed two steps, which is the C_α_–H bond cleavage and hydride transfer to the N5 atom of LFN (see Scheme 1), we computed the IRC reaction profile in the gas phase and in the SCRF starting from the TS obtained from relaxed potential energy surface scans using the distance between the migrating hydrogen and N5 atom of LFN as a control variable. Gas-phase geometries acquired in the IRC scan were then used to compute frontier molecular orbitals of the PEA and LFN molecule and their respective energies; in these calculations, the two molecules were treated separately.

Explicit solvation models were constructed from the precedent simulation of reaction dynamics in explicit aqueous solution,^20,21^ by taking several snapshot structures from that simulation and reducing the size of the system to the PEA···LFN moiety and few (9–15) nearest water molecules. Such clusters contain up to 96 atoms and can be reasonably treated with presently employed quantum chemistry protocols. In all these clusters, the PEA···LFN moiety was subject to relaxed potential energy surface scans either by (i) displacing the migrating hydrogen (hydride) between the C_α_ atom of PEA and N5 atom of LFN or (ii) varying the distance between C_α_ and N5. The first of the two strategies was used to generate starting geometries for TS optimization of the hydride transfer step. After TS optimization, IRC profiles were computed in both directions as described above. In total, 12 such profiles were obtained for the explicitly solvated reacting system.

The second set of scans (case (ii)) facilitated the search of the minimum-energy structures of the intermediate complex, both in a covalently bound variant featuring the C_α_–N5 bond, as well as in a form of ionic pair with longer C_α_···N5 separation. Several successive relaxed scans along the C_α_···N5 line in both directions were performed (elongating the C···N separation, then shrinking it, elongating it again, and so forth). The C···N distance range spanned by the scans was roughly between 1.6 and 4.2 Å. All the minima found on the so-obtained potential energy functions were then optimized without restrictions, using both the gas-phase model as well as implicit solvation (SCRF). After eliminating duplicates, a total of 42 optimized structures of the intermediate complex in the gas phase and 35 optimized structures in the implicit solvent were collected.

The final step required to complete PEA oxidation is the hydrogen/proton transfer from the amino group of PEA to the N1 atom, yielding the corresponding imine and reduced flavin (hydrogenated at both the N1 and N5 positions; see Scheme 1). In order to facilitate hydrogen transfer, one or two bridging water molecules were optionally placed between the hydrogen-donating amino group of PEA and the N1 acceptor of LFN. Hydrogen transfer was enforced by a relaxed potential energy scan using one of the involved N···H and O···H distances as control variables, and attempts were made to find a proper TS for this process. This step was also considered in various variants, including the gas phase and implicit solvation model, as well as in the presence of explicit water molecules, as described above.

## 3 Results and Discussion

### 3.1 C–H Bond Cleavage and Hydride Transfer in the Gas Phase

**Figure 3:**
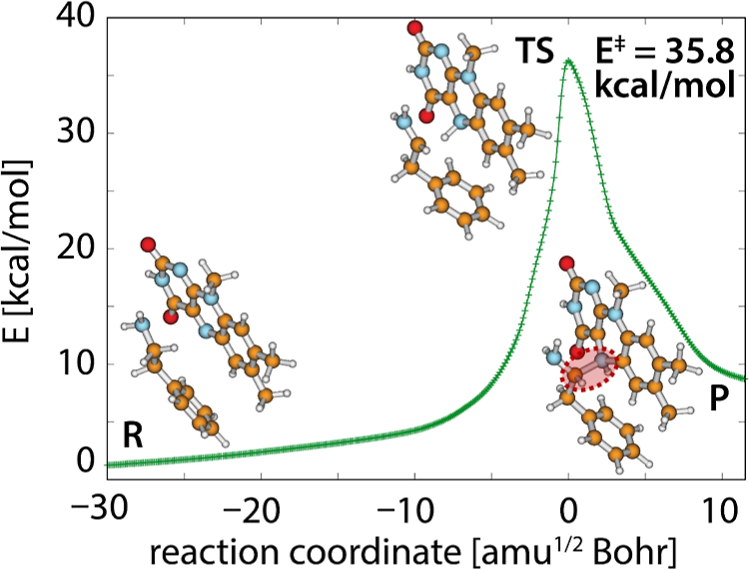
Computed IRC profile of the hydride transfer step in the gas phase together with structures corresponding to reactants (R), TS, and products (P). Note that the product state corresponds to the intermediate complex discussed in the text. In the product state, the covalent bond formed between the C_α_ atom of PEA and the N5 atom of LFN is emphasized by a red-shaded ellipse with a dashed outline.

While this step has been explicitly investigated in the past,^19^ and its selected parts have been used for the modeling of MAO reactions,^20,44^ the present expanded treatment includes the IRC profile displayed in Figure 3. In agreement with previous calculations, the gas-phase IRC profile ends at both sides with regular minima representing the prereaction complex on the reactant side and the intermediate complex on the product side (Figure 3). The barrier deduced from IRC calculations amounts to 35.8 kcal/mol, which is in fine agreement with previous similar treatments.^20,44^ The products of this step are 6.9 kcal/mol above the reactants. At the reactant (R) side, the profile appears to be quite flat and stretched, which corresponds to a large conformational flexibility of the PEA···LFN complex in a nonbonded state. In contrast to that, the product (P) (intermediate) complex is rather stiff because of the covalent C_α_–N5 bond formed between the two entities, which is reflected in a relatively steep profile at the product side.

**Figure 4:**
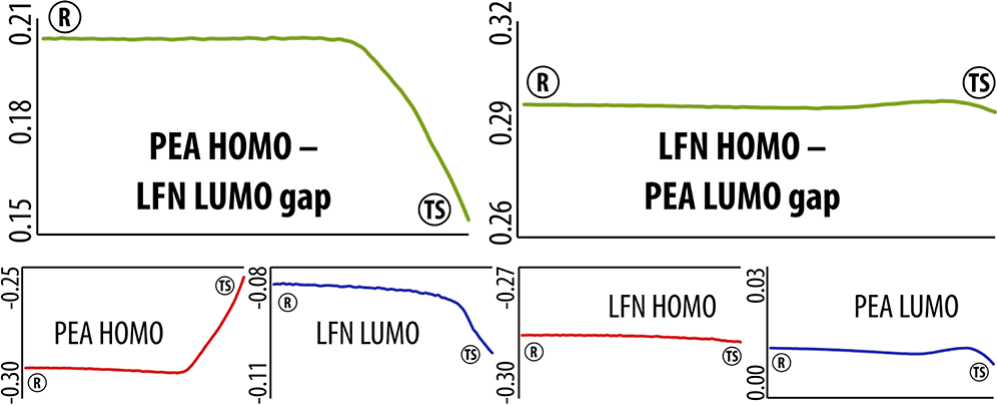
HOMO–LUMO gap as a function of IRC together with functional dependence of individual frontier orbitals for both directions of charge transfer. The *x* axis represents the IRC coordinate in the range between the reactant (R) and the TS, whereas the *y* axis is the (orbital) energy in au.

Using geometries acquired along the IRC pathway, we computed the frontier molecular orbitals and their energies. This was done separately for both PEA and LFN, yielding the HOMO–LUMO gap as a function of the IRC coordinate. Both directions of electron flow were considered by taking either HOMO of PEA and LUMO of LFN (negative charge transfer from PEA to LFN) or vice versa for the opposite direction of charge transfer. The HOMO–LUMO gap profiles are displayed in Figure 4 for both scenarios. Please note that evaluation of the frontier orbitals has been done only for the uphill part of the pathway, i.e., from R to TS, because on exchange of hydrogen between PEA and LFN that follows shortly after the TS, the definition of both entities changes, impairing energy comparison of the orbitals.

The profiles clearly demonstrate the significant preference of negative charge transfer from PEA to LFN, in that the corresponding HOMO–LUMO gap is (i) significantly narrower than in the opposite case and (ii) exhibits reasonable dependence on the IRC coordinate, including noticeable shrinking on approaching the TS. In contrast to that, the HOMO–LUMO gap for charge transfer in the reverse direction is by ∼50% larger and virtually independent of the reaction coordinate. This confirms the nature of this step to be a hydride transfer. Visualization of the HOMO of PEA fully supports this, in that on approaching the TS, the orbital becomes localized at the elongating C–H bond and its vicinity (see the Supporting Information, Figure S1), indicating the principal area of reactivity.

### 3.2 Effects of Solvation on Hydride Transfer

Implicit solvation (SCRF) has only little influence on either the reaction profile or the structure of the involved entities. The shape of the profile barely changes relative to the gas phase, but both the barrier and the reaction energy are reduced by about 4 to 31.7 and 2.7 kcal/mol, respectively. This appears to be in agreement with the fact that the polarity of the reacting moiety steadily increases during this reaction step.^26^ The unusual C_α_–N5 bond persists but is additionally elongated from 1.62 to 1.65 Å. Apart from this slight elongation, no evidence of the weakening or cleavage of the C_α_–N5 bond could be devised from SCRF calculations. All attempts at obtaining an unbound intermediate complex of positively charged PEA and negatively charged LFN entities failed at this level of modeling.

Explicit solvation changes this quite drastically, in that it facilitates formation of the intermediate in the form of an ion pair featuring C_α_···N5 separation much beyond covalent bonding. We performed several evaluations of the reaction profile in the presence of 9–15 explicit water molecules, and the results are summarized in Table 1.

One of the principal features of reaction barriers and energies computed for a variety of microsolvated models is their perceivable fluctuation: the barrier is in the range between 27 and 40 kcal/mol, whereas the energy varies between −7 and +7 kcal/mol. These variations can be attributed to the limited size model and mainly reflect the large conformational flexibility of the network of water molecules and their interactions. The variations are roughly equivalent to the energy of two hydrogen bonds (H-bonds) formed between two water molecules or between a water molecule and one of the functional groups capable of donating or accepting an H-bond (e.g., the amino group of PEA or N1 of LFN). Since the structure of the water network changes substantially along the reaction profile, formation or cleavage of one or two H-bonds can readily occur, which explains the observed variations in the reaction barrier and energy. But, in order to get meaningful information from such calculations, one needs to consider a sufficiently large sample of such solvated models to at least partly reflect the myriad of possible conformations of the surrounding water molecules on account of their conformational flexibility. The strategy of acquiring several models as described in Section 2 is aimed at obtaining a statistically relevant set of representative structures. For the same reason, we used a similar approach also in our investigation of the C_α_–N5 bond dissociation, as will be presented in Section 3.3. While the relatively high computational requirements of Hessian-based techniques such as TS optimizations and particularly IRC calculations prevented us from generating more than a dozen profiles, we feel that the present results quite reasonably assess the influence of solvation. The average barrier of 33.2 kcal/mol is barely different from the one computed by the implicit solvation methodology and is in agreement with previous studies.^21,26^

**Figure 5:**
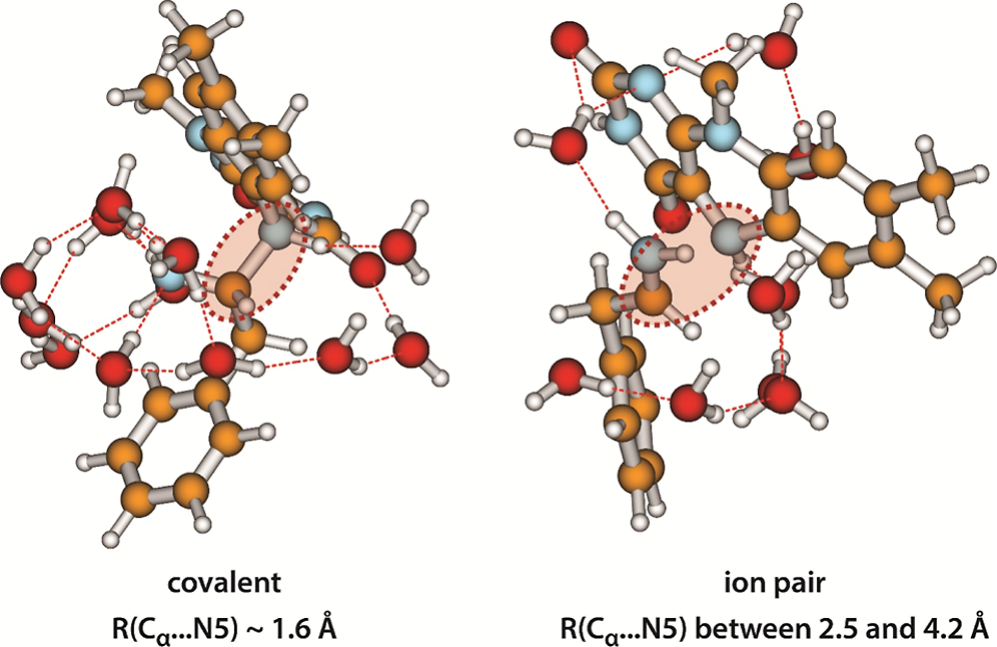
Two characteristic examples of the intermediate complex formed by semioxidized PEA and semireduced LFN, as predicted by calculations involving explicit water molecules. Left: a covalently bound complex. Right: an ion pair. The C_α_–N5 moiety is marked by a shaded ellipse with a dashed red outline.

Variations in the geometry of products, i.e., of the intermediate complex consisting of a partially oxidized PEA and partially reduced LFN molecule, provide valuable new insight into this reaction. Namely, unlike all previous studies, IRC calculations predict the formation of a covalent C_α_–N5 bond only in a minor part (2 out of 12) of explicitly solvated models, whereas in the majority of cases, the LFN···PEA complex remains in an unbound state with the C···N separation of 2.5 Å or larger, thereby constituting a solvated ion pair. Two characteristic structures of the intermediate complex are displayed in Figure 5, and several other structures of entities associated with the data in Table 1 are presented in the Supporting Information, Figure S2. An in-depth investigation of factors governing the existence of the covalent C_α_–N5 bond and its dissociation will be presented in Section 3.3.

Worthy to note is that unlike the intermediate state, variations in TS geometry of the C_α_···H···N5 moiety are almost nonexistent between the models, the average C···N separation being about 2.55 Å, with individual offsets barely exceeding ±0.02 Å. Likewise, hydrogen is in all cases located almost equidistantly between C_α_ and N5 but slightly closer to N. Almost the same TS geometries have also been observed in the gas phase and with the SCRF model. The relatively stiff TS structures are quite common in various chemical processes, including proton transfer in H-bonds,^45^ and the stiffness of the TS has also been observed for this reaction in the course of reaction dynamics simulation using classical mechanics and empirical force fields.^22^

Importantly, in all of the cases, the imaginary mode of the TS only includes displacements within the C_α_···H···N5 moiety, yielding no evidence for potential coupling of hydride transfer with other chemical transformations (such as, for example, proton transfer from the amino group of PEA to the N1 atom of LFN). This means that a concerted reaction mechanism comprising hydride transfer and some other process appears to be unlikely. All the computed IRC profiles fully support this observation. Together with the occurrence of a stable intermediate (either in the form of a covalently bound complex or an ion pair), this provides evidence that the reaction proceeds in at least two well-separated steps.

### 3.3 C_α_–N5 Bond and Dissociation of the Intermediate Complex

**Figure 6:**
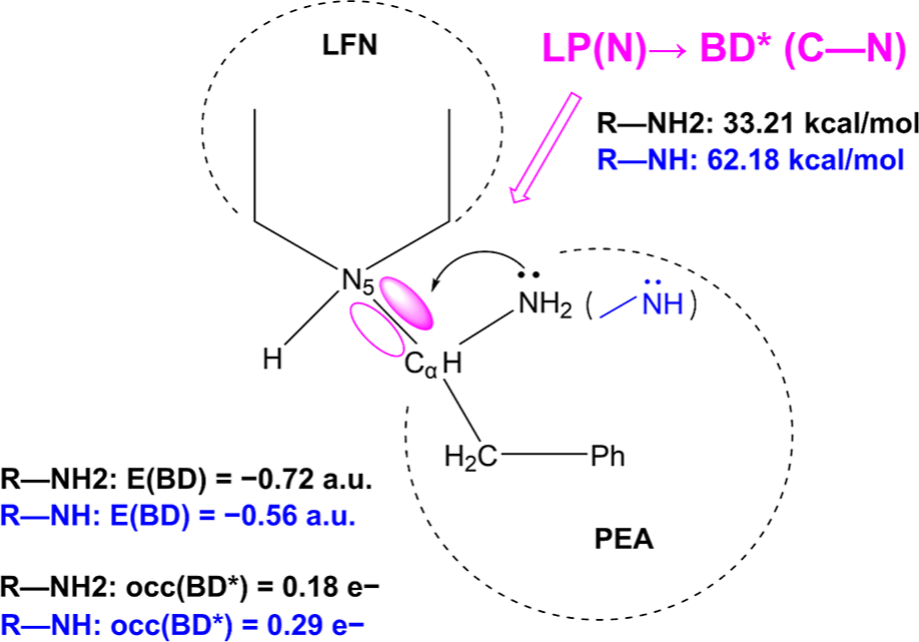
Electronic effects governing the increased length of the C_α_–N5 bond in the intermediate complex, as computed by NBO analysis. Characteristic orbital features (see text) are listed for both neutral (–NH_2_) and deprotonated (–NH) amino groups in black and blue color, respectively. The donor–acceptor lone pair-antibonding orbital weakening the C_α_–N5 bond is shown in pink. The PEA and LFN entities constituting the system are marked with a dashed line.

The C_α_–N5 bond was investigated by an NBO analysis. The following four quantities appear to be particularly indicative of the stability of the C_α_–N5 bond: (i) energy of the C_α_–N5 bonding orbital; (ii) its occupancy; (iii) interaction between the lone-pair orbital located at the amino nitrogen and the C_α_–N5 antibonding orbital, with the former being the donor and the latter the acceptor of electrons; and (iv) population of the C_α_–N5 antibonding orbital. These quantities (except for (iv)) are schematically presented in Figure 6. The C_α_–N5 bonding orbital stands out as the highest in energy among all C–N bonds present in the system (−0.72 au, as compared to others ranging between −0.82 and −0.94 au); it is also lowest in occupancy (1.971 electrons). At the same time, the C_α_–N5 antibonding orbital features a noticeable population of 0.182 electrons, and there exists a strong donor–acceptor interaction of 33.21 kcal/mol between the lone-pair orbital on the amino nitrogen and the C_α_–N5 antibonding orbital, with the former being the donor and the latter the electron acceptor. The relatively high energy of the bonding orbital together with the substantially populated antibonding orbital and its strong interaction with an electron donating orbital suggest a considerable weakening (lengthening) effect on the C_α_–N5 bond. The electronic “pathway” from amino nitrogen to the C_α_–N5 antibonding orbital is likely the main source of the surprising bond length (Figure 6).

Furthermore, the lengthening of the C_α_–N5 bond is likely coupled with the dissociation of a proton from the amino group, which is an essential step for completing the reaction (yielding the final imine and dihydrogenated flavin products). This has been demonstrated by performing NBO analysis on the variant of the optimized intermediate complex in which a proton was removed from the amino group while the rest of the system was kept in the same geometry. Figure 6 displays a comparison of the aforementioned NBO results between the neutral (R–NH_2_) and deprotonated (R–NH) PEA moiety of the complex. When the proton is removed from the amino group, the C_α_–N5 bonding orbital energy significantly increases (from −0.72 to −0.56 au) and so does the population of the corresponding antibonding orbital (from 0.182 to 0.286 electrons; not shown in Figure 6). The interaction energy between the amino nitrogen lone pair and the antibonding orbital nearly doubles (from 33.21 to 62.18 kcal/mol). All of this suggests that the C_α_–N5 bond is further weakened upon departure of the proton from the amino group; indeed, optimization of such complex results in spontaneous cleavage of the C_α_–N5 bond and the complex disintegrates. It may be concluded that the existence of the unusual C_α_–N5 bond is due to the fact that the intermediate complex corresponds to an incomplete oxidoreduction process in which the reacting entities PEA and LFN exist in a semioxidized and semireduced state, respectively. In this complex, the PEA entity features a peculiar electronic structure: (i) a carbocation formed at C_α_ by the hydride abstraction, with a tendency to fill the valence vacancy by forming a C_α_–N5 bond with LFN, but, just the opposite to that, (ii) PEA retains a lone electron pair on the vicinal amino nitrogen with a strong tendency of forming a double bond with C_α_, rendering the C_α_–N5 bond less stable (and, upon deprotonation of the amino group, impossible). The observed C_α_–N5 bond length is likely due to the balance of these opposing factors.

**Figure 7:**
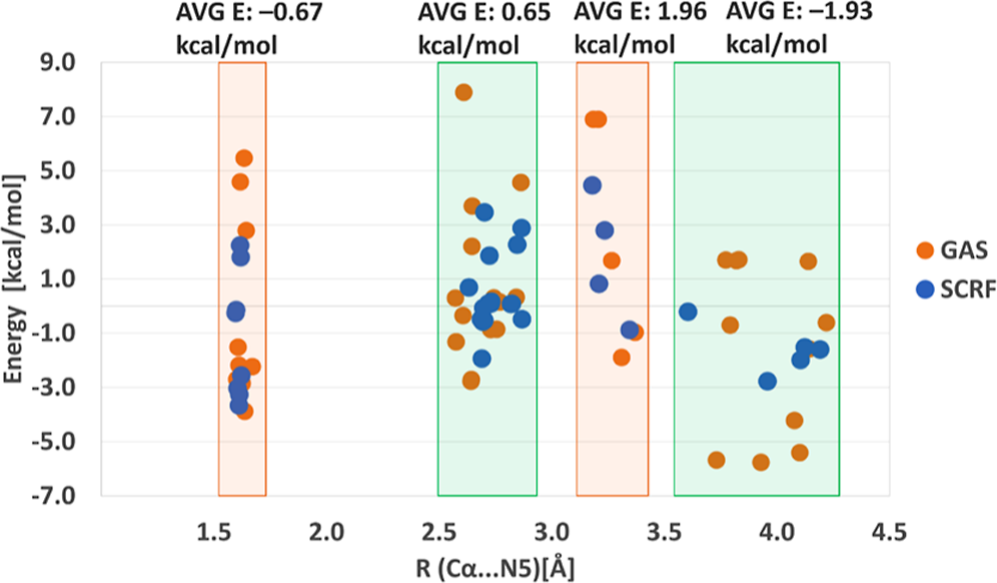
Relative energies of minimum-energy structures of the PEA···LFN intermediate complex explicitly solvated by 9–15 water molecules, as a function of the C_α_···N5 distance (see Figure 5 for representative structures). The structures were obtained by several successive relaxed potential energy surface scans along the C_α_···N5 distance in both directions. Note that the models include different numbers of water molecules, and for each distinct model, the energy is given relative to the average energy of all the minima found for that model. The four distinct regions of stable structures are highlighted by colored frames, and the average energy within each region is displayed on top. Results obtained by gas-phase calculations are shown as orange dots whereas those computed by the implicit solvation model (SCRF) are displayed in blue.

To further elucidate interactions between (semioxidized) PEA and (semireduced) LFN in the intermediate complex, we used, similar to the above for the hydride transfer step (Table 1), a series of explicitly solvated models including 9–15 water molecules. By performing successive forth and back relaxed scans along the C_α_···N5 distance, we found several energy-minimum structures differing in geometry and energy. Figure 7 shows the energies of these structures as a function of the C···N separation.

Large variations in the optimized C_α_···N5 distance demonstrate that oppositely charged PEA and LFN entities can coexist at nonbonding distances in the presence of just a few water molecules. According to the clustering of points in Figure 7, stable ion pairs can be found at C···N distances of about 2.7, 3.3, and 4.0 Å. Most of these ionic pairs are barely different in energy from that of the covalently bound PEA···LFN complex (the cluster located at ∼1.6 Å). Specifically, the average energy of minima in these clusters amounts to 0.7, 2.0, and −1.9 kcal/mol, respectively, relative to the average energy of all the minima. Likewise, the average minimum energy corresponding to the covalently bound complex at C···N separation of ∼1.6 Å is approximately −0.7 kcal/mol. According to the potential energy surface scans performed over the 1.6–4.2 Å range, the maxima separating these minimum energy regions are in the range of 3 kcal/mol, indicating that any TS related to dissociation of the PEA···LFN complex is low enough in energy not to interfere with the rate-limiting step. The explicitly solvated models were optimized both in the gas phase as well as with the SCRF approach, but no significant difference between the gas phase and SCRF cluster model could be found (Figure 7), indicating that just a few nearest water molecules in the vicinity of the reacting moiety are sufficient to account for a large part of the solvation effect. Similarly to the hydride transfer step modeled in the presence of explicit water molecules, energy variations between the minima are up to about 12 kcal/mol. This energy span is approximately equivalent to the energy of two H-bonds established between water molecules or between water molecules and the PEA···LFN complex and is consistent with the observed changes in the H-bonded network during the potential energy surface scans.

While the detailed study of the energetics of the C_α_···N5 bond dissociation has been performed using between 6 and 15 water molecules (Figure 7), tentative attempts have been made at determining the minimum amount of explicit water molecules surrounding the intermediate PEA···LFN complex that still supports the existence of the ion pair. By repeatedly removing water molecules, one at a time, from the previously optimized intermediate structures listed in Table 1 and Figure 7, and optimizing the complex in the presence of a reduced number of water molecules, we have been able to obtain a stable ion pair complex surrounded by only four water molecules. We take the number four to be an estimated upper limit of the minimum requirement of explicit water solvation to obtain a stable intermediate in a dissociated form (note that energetics of dissociation has not been undertaken in this evaluation). At this point, considering that only four water molecules can facilitate the formation of a PEA···LFN ion pair, it can be deduced that the active site of MAO enzymes also likely facilitates dissociation of the intermediate complex, despite the fact that in both MAO-A and MAO-B, the active site is generally considered as hydrophobic. While the abundance of aliphatic and aromatic residues supports the perception of a hydrophobic active site, it should be noted that aromatic rings of tyrosine and phenylalanine are electric quadrupoles and are capable of establishing polar interactions with the substrate or intermediate (we noticed such interactions between the substrate and enzyme in our previous simulations of MAO reactions).^22^ In addition, tyrosine residues can directly participate in hydrogen bonds with their OH groups. Also, few polar and even one charged residue (lysine) exist in the MAO active sites. Therefore, several residues in the vicinity of the reacting moiety can possibly stabilize a charged (ion pair) intermediate complex. Among those, two tyrosines forming the so-called “aromatic cage” of MAO active sites^46,47^ are in a particularly favorable location for this purpose. The assumption that the MAO active site is less hydrophobic than it may look at first glance is also supported by EVB simulations of reaction dynamics, in which ∼5–7 water molecules are consistently present at a distance of less than 7 Å from the reacting moiety.^20,22^ Therefore, it can be assumed that the ion pair intermediates can likely be sufficiently stabilized even by the (presumably hydrophobic) enzymatic environment, which is in full agreement with the findings of Maršavelski and Vianello for the oxidation of HIS and NMH by MAO-B.^31^

In all variants of the intermediate complex, a negative charge flow from PEA to LFN is confirmed, and the amount of transferred charge increases with the increasing C_α_···N5 separation. For covalently bound complexes with a C_α_–N5 bond of ∼1.6 Å, charge transfer (derived from natural atomic charges, which are computed within NBO analysis) is just below 0.4 electrons, but for ionic pairs, it increases to about 0.75 electrons at a C_α_···N5 separation of ∼2.6 Å and to nearly 0.9 electrons at a separation of ∼4.0 Å. This is fully consistent with the ionic nature of the constituents of the intermediate complex and in agreement with the presumed charge flow supported by analysis of frontier molecular orbitals (see Section 3.1). This additionally confirms the hydride transfer nature of the reaction.

The influence of surrounding water molecules on dissociation of the PEA···LFN complex was also examined by NBO analysis. We investigated 13 minimum-energy structures corresponding to the covalently bound complex (C_α_···N5 distance of ∼1.6 Å), all in the presence of explicit water molecules, focusing on the aforementioned parameters indicative of C_α_–N5 bond weakening, as presented above (see Figure 6). This time, we monitored the change in NBO quantities on removal of the surrounding water molecules while keeping the geometry of the PEA···LFN moiety unchanged. With only minor exceptions that are of little relevance, the C_α_–N5 bond weakening effect is reduced by all criteria for removal of water molecules in practically all cases. Specifically, the C_α_–N5 bonding orbital energy decreases and its occupancy increases. At the same time, the interaction energy between the lone pair on amino nitrogen and the C_α_–N5 antibonding orbital decreases and so does the occupancy of that antibonding orbital. This leads to the conclusion that interaction of the PEA···LFN complex is assisted at the electronic structure level by interactions provided with surrounding water molecules. Further details on this analysis are given in the Supporting Information, Table S1.

The fact that the solvated intermediate complex can exist in various conformations differing greatly in geometry but barely in energy can possibly explain the reportedly different intermediate structure in the active site of MAO-B between dopamine on one side and HIS and NMH on the other, as predicted by quantum calculations.^19,31^ Namely, the intermediate complex involving dopamine is covalently bound,^19^ whereas the one with HIS and NMH is preferably dissociated.^31^ In both cases, the explicit enzymatic environment consisting of a few residues and water molecules has been included in the model. Since even a small variation in simulation conditions, not only the number and position of surrounding water molecules but most likely also the size and chemistry of the amine substrate, the presence of amino acid side chains, etc., can cause substantial changes in the structure of the intermediate, similar variations can be expected in studies involving explicit protein surroundings. While the assumption of a different chemistry between dopamine and histamine being the cause of the observed differences appears to be reasonable,^31^ these differences are in full agreement with our results, suggesting that the related potential energy surface is rather flat, supporting large geometry variations at small energy costs. Also, our calculations of the gas-phase HIS···LFN and NMH···LFN covalent complex yield a virtually identical C_α_–N5 distance of 1.61 Å both for HIS and NMH, giving no evidence of a sizable difference between HIS or NMH from PEA, let alone of any C_α_–N5 bond weakening due to the different chemistry of HIS and NMH. This further suggests that the shallow potential energy surface may be the main cause of the large structural variations of the intermediate with different amine substrates, even within the enzyme active site.

### 3.4 Reaction Completion by Proton Transfer

The final step of amine oxidation by flavin is the conversion of the semioxidized amine substrate to imine by deprotonation of the amino group. At the same time, flavin is converted from the semireduced into the fully reduced (dihydrogenated) form by binding the departed proton to the N1 ring atom. As our investigations of the hydride transfer step and of the stability of the intermediate complex yielded no evidence of a concerted process involving both hydride and proton transfer, we conclude that proton transfer from the amino group of PEA to the N1 atom of LFN proceeds as a separated step.

Our studies of the proton transfer step start at the isolated intermediate complex featuring a covalent C_α_–N5 bond, attempting to find a reasonable proton transfer pathway from amino nitrogen to N1. As it may be intuitively expected, no TS could be found for this process. The relaxed potential energy function for proton transfer between the corresponding atoms features a maximum at ∼29 kcal/mol in the gas phase. This suggests that a direct proton transfer from N(PEA) to N1(LFN) is unfavorable. However, inclusion of one or two explicit water molecules that bridge the space between the amino group of PEA and N1 of LFN results in a substantial lowering of the potential energy profile and the corresponding TS can easily be found at ∼15–16 kcal/mol above the energy of the intermediate complex. The TS includes a compressed H-bond between the amino group and the bridging water molecule, with the N···O separation slightly below 2.5 Å and the proton located close to the midpoint but nearer to the acceptor oxygen atom. At the same time, the C_α_···N5 distance is elongated to over 2.6 Å. Inspection of geometries involved in the IRC profile for the uphill part reveals that in the first part of the process, the C_α_–N5 bond cleavage is predominant, with the C_α_···N5 distance increasing from ∼1.6 to ∼2.5 Å. At the same time, the H-bond involving the amino group and the bridging water molecule shrinks but to a much lesser extent (from ∼3.0 to ∼2.7 Å), whereas the proton remains firmly at the amino group with yet little if any tendency of migrating over the H-bond. The energy cost of this part of the process is estimated to be ∼8 kcal/mol (see the Supporting Information, Figure S3), comprising about half of the barrier height, and it can be deduced that this part of the barrier is mainly due to the requirement of breaking the C_α_–N5 bond. Because this bond can be cleaved at little if any cost in the presence of explicit water molecules, the proton transfer barrier is expected to be lower for the explicitly solvated model, as will be demonstrated below.

Given that the gas-phase energy of the intermediate complex is ∼ 7 kcal/mol above the energy of reactants (Table 1), the proton transfer barrier is at roughly 22–23 kcal/mol above reactants, meaning that the TS of the proton transfer is by at least 12 kcal/mol below the TS pertaining to the precedent hydride transfer. This renders the possibility that the proton transfer step interferes kinetically with the hydride transfer step highly unlikely. In the solution, in which the intermediate complex is more stable relative to the gas phase (Table 1) this is even less probable, suggesting that the proton transfer step features considerably lower TS energy than the hydride transfer step.

We also evaluated the barrier of the proton transfer step by an explicitly solvated model. In contrast to the hydride transfer step, we considered only one configuration of the surrounding water molecules. Among many available structures of the explicitly solvated intermediate complex, we picked one with a bridging water molecule present between the amino group of PEA and the N1 atom of LFN (entry #8 in Table 1) and used that structure to perform TS search followed by IRC profile calculations. A regular TS has been found, and the computed barrier of 8.1 kcal/mol is considerably lower than in the absence of surrounding water molecules. This can be readily explained by the fact that owing to solvation, the complex already exists in a dissociated form with a C_α_···N5 separation of ∼3.5 Å; hence, in contrast to the isolated model, no energy input associated with C_α_–N5 bond dissociation is required. From the isolated model, we estimated the energy input required for dissociation to be ∼8 kcal/mol, which is consistent with the almost double barrier height associated with that model (see the Supporting Information, Figure S3).

As the intermediate is already at 4.2 kcal/mol below the reactant state for model #8 (Table 1), the proton transfer TS obtained from the explicitly solvated model is therefore located at −4.2 + 8.1 = 3.9 kcal/mol relative to the reactant state, thus being by over 25 kcal/mol lower in energy than the TS of hydride transfer, a difference exceeding maximum variations in the energy due to fluctuations of the cluster of water molecules (Table 1) by a factor of 2. Consequently, the TS of the proton transfer step predicted by the explicit solvation model is extremely unlikely to be of comparable or higher energy than the TS of the hydride transfer step. As such, this step is of little relevance for the reaction kinetics; therefore, we refrained from evaluating the proton transfer energy profiles on more than one structure. All in all, this provides convincing evidence that the hydride transfer step is indeed rate-limiting for the reaction in question.

**Scheme 2:**
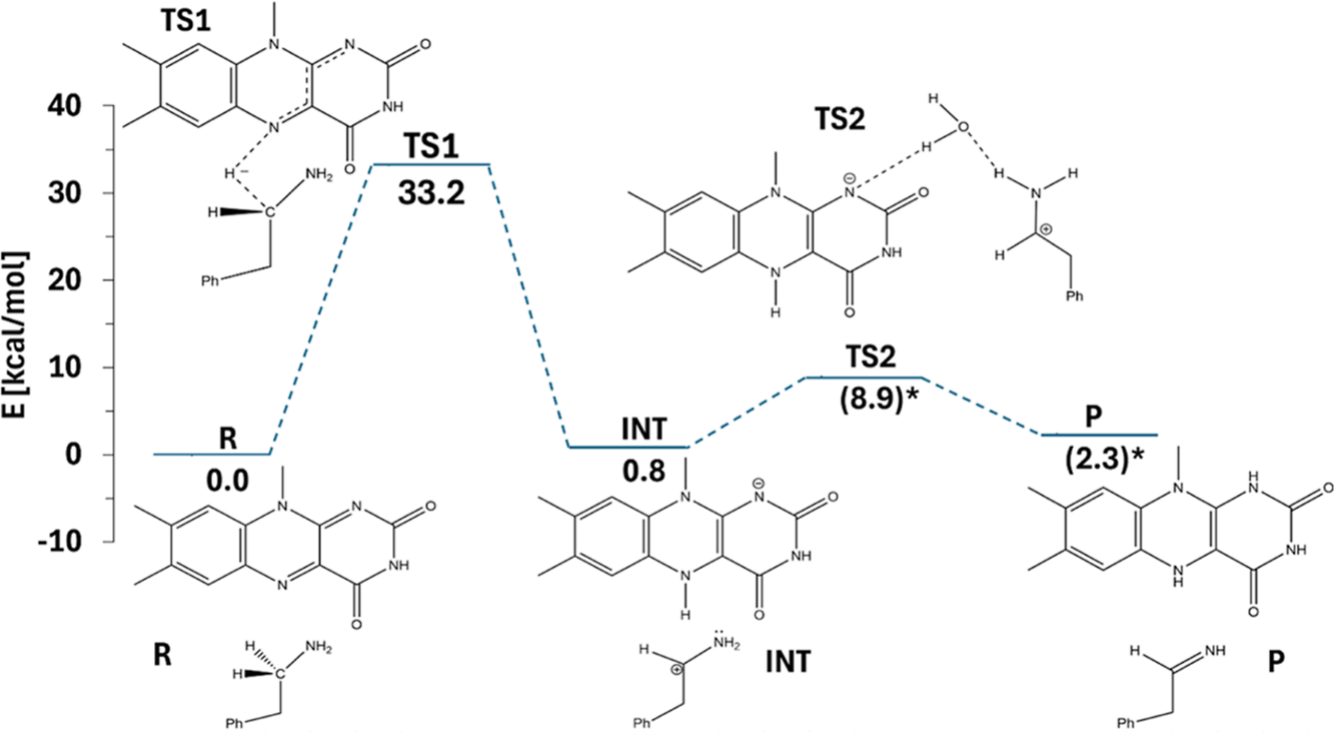
Energetics of Amine Oxidation by Flavin Proceeding by the Two-Step Hydride Transfer Mechanism Explicit water molecules undertaken in the calculation are not shown unless they take an essential part of the mechanism, i.e., by providing a proton transfer bridge. Values in parentheses indicate energy evaluation using a single structure rather than averaging over a number of explicitly solvated structures.

Summarizing the characteristics of PEA oxidation by LFN, the reaction mechanism is schematically presented in Scheme 2. The explicitly solvated variant is chosen because it likely represents the real situation most faithfully among the models.

## 4 Conclusions

The present study provides in-depth computational insight into amine oxidation cast by flavin, a reaction of paramount importance for the metabolism of monoamine neurotransmitters. Focusing on selected aspects of the mechanism that have not been fully elucidated to date, the hydride transfer nature of the mechanism has been confirmed by the analysis of frontier molecular orbitals and the evaluation of charge transfer. While both the gas-phase and SCRF treatment of an isolated reacting system yield reasonable barriers of 32–36 kcal/mol and energies of 3–7 kcal/mol for the hydride transfer step, they do not deliver a complete explanation of the reaction nor of the structure of the reactive intermediate, in that they consistently predict an unusually long (∼1.62 Å) C_α_–N5 bond connecting the involved PEA and LFN entities, which barely exhibits any dissociation tendency. In fact, the relatively demanding energetics of dissociation of the C_α_–N5 bond has left the question of the rate-limiting step open. In contrast to calculations on isolated models (be gas phase or SCRF), explicit solvation models clearly demonstrate that in a polar environment, dissociation of the intermediate is possible at only little cost; in fact, the intermediate is likely to be formed as an ion pair during the hydride transfer step without any covalent interaction between the entities. Nevertheless, the PEA···LFN intermediate was found to be stable at any condition, implying the reaction to proceed in at least two distinct steps. While having a considerable effect on the structure of the intermediate, explicit solvation delivers an average barrier and reaction energy of 33.2 and 0.8 kcal/mol, respectively, which is barely different from the SCRF model.

The rather unusual nature of the C_α_–N5 bond has been examined by the NBO analysis of the electronic structure. The bond possibly exists at such a high length due to the carbocation nature of C_α_ resulting from abstraction of a hydride ion, whereas at the same time, the amino group remains intact because the intermediate state corresponds to an incomplete oxidoreduction process. The C_α_ carbocation is involved in two competing interactions available for filling up its vacant valence orbitals, one with the N5 atom of LFN and the other with the lone pair at the amino group of PEA. The former promotes the formation of the C_α_–N5 bond, whereas the latter disrupts it; the abnormal length is likely a consequence of the two opposing factors. The electron lone pair at the amino nitrogen features a strong tendency of delocalizing into the C_α_–N5 antibonding orbital, thereby weakening the bond; in addition, both deprotonation of the amine group as well as interactions with surrounding water molecules also enhance disruption of the C_α_–N5 bond by this mechanism. This explains the feasibility of C_α_–N5 bond dissociation on inclusion of explicit solvent molecules in the model. The low number of (four) water molecules found to be sufficient to facilitate dissociation, together with the capability of certain residues of the active site of MAO enzymes to establish polar interactions with the substrate and the intermediate complex, support the assumption that the reaction intermediate can exist in a dissociated form also in the active site of MAOs, which is in full agreement with a previous DFT study of histamine oxidation in the active site of MAO-B.^31^ Also, the previously observed difference between dopamine^19^ and histamine^31^ in their tendency of intermediate dissociation is largely supported by the presently elucidated energetics of intermediate dissociation, in that polar interactions with the surroundings facilitate dissociation at a small energy cost, meaning that the potential energy surface related to dissociation is rather shallow.

Following the hydride transfer step, the reaction is completed by the migration of the amino hydrogen to the N1 atom of LFN, yielding PEA oxidized to imine and fully reduced (dihydrogenated) LFN. Calculations show that at least one bridging water molecule is required to facilitate this process. Also, the IRC profile reveals that dissociation of the C_α_–N5 bond to a separation of ∼2.5 Å is a prerequisite for proton transfer, requiring an input of ∼8 kcal/mol just to bring the system to the onset of proton transfer. Consequently, explicitly solvated models in which the PEA···LFN complex already exists at sufficient C_α_–N5 separation feature a much lower barrier of ∼8 kcal/mol, as opposed to 15–16 kcal/mol for nonsolvated models. In any case, the barrier associated with the proton transfer step is significantly lower than the one corresponding to hydride transfer, confirming the latter as rate-limiting. Importantly, none of the evaluated reaction pathways or transition states gives any evidence for the hydride and proton transfer to occur in a concerted manner, thereby validating the mechanism consisting of two distinct steps.

This work demonstrates that explicit interactions between the reacting system and its (polar) environment have a notable effect on relevant parameters of the reaction. As the reaction in question occurs in the active site of MAO enzymes, it would be a challenge to properly assess the specific interactions established between the reacting moiety and the surrounding residues. The quantum chemical study of Vianello et al.^19^ partly addressed this issue by including three relevant tyrosine residues and four water molecules in their models. While the computed hydride transfer barrier was significantly lower than in the gas phase or in implicit solvent, namely, 24–26 kcal/mol depending on the substrate, the covalent C_α_–N5 bond appears to persist in the intermediate complex. Here, Vianello’s study neatly elucidates the catalytic function of MAO enzymes and discerns between several proposed mechanisms; however, it somewhat leaves aside details such as the stability and role of the presently investigated C_α_–N5 bond. We feel that for proper assessment of this aspect, a more complex model would be needed, including several residues and more water molecules. However, such a model would likely push the required computational resources to their limits. Nevertheless, the herein devised correction of the mechanism based on dissociated reacting entities in the intermediate state appears to be a viable option also in the enzyme active site.

## Supporting Information Available

Visualization of frontier molecular orbitals, explicitly solvated intermediate models, and selected IRC pathways; detailed results of selected NBO calculations; and IRC profiles for the proton transfer step in the gas phase and in the presence of explicit water molecules (PDF)
Optimized structures of entities reported in this study and geometries along the IRC pathway (TXT)
