# Correction to “Deciphering the Two-Step Hydride Mechanism of Monoamine Oxidase Flavoenzymes”

**DOI:** 10.1021/acsomega.6c04558

**Published:** 2026-06-08

**Authors:** Martina Rajić, Alja Prah, Jernej Stare

**Affiliations:** † Faculty of Pharmacy, University of Ljubljana, Aškerčeva cesta 7, 1000 Ljubljana, Slovenia; ‡ Theory Department, Laboratory for Computational Biochemistry and Drug Design, National Institute of Chemistry, Hajdrihova 19, Ljubljana SI-1000, Slovenia

The authors wish to correct the published article with the following changes:

1. One of the two affiliations of Martina Rajić was omitted in the original publication. The additional affiliation must be listed as the primary affiliation and should read: Faculty of Pharmacy, University of Ljubljana, Aškerčeva cesta 7, 1000 Ljubljana, Slovenia. The existing affiliation of Martina Rajić (Theory Department, Laboratory for Computational Biochemistry and Drug Design, National Institute of Chemistry, Hajdrihova 19, Ljubljana SI-1000, Slovenia) should remain unchanged.
2. Additionally, the ORCID identifier for Martina Rajić was not included in the original publication.

These corrections are reflected in the Author Information of this Correction. They do not affect the scientific conclusions of the paper.

