# Supplementary material for: Deciphering the Two-Step Hydride Mechanism of Monoamine Oxidase Flavoenzymes
Source: ACS Omega. 2024 Oct 10;9(42):43046–57. doi: 10.1021/acsomega.4c06575 (PMC11500147; doi:10.1021/acsomega.4c06575)
Supplement: Supplementary file 1 — ao4c06575_si_001.pdf [file ao4c06575_si_001.pdf]

Deciphering the Two-Step Hydride Mechanism of Monoamine Oxidase Flavoenzymes

*Martina Rajić, Alja Prah, Jernej Stare**

Theory Department, Laboratory for Computational Biochemistry and Drug Design

National Institute of Chemistry, Hajdrihova 19, SI-1000 Ljubljana, Slovenia

**Corresponding author, e-mail: jernej.stare@ki.si*

SUPPLEMENTARY INFORMATION

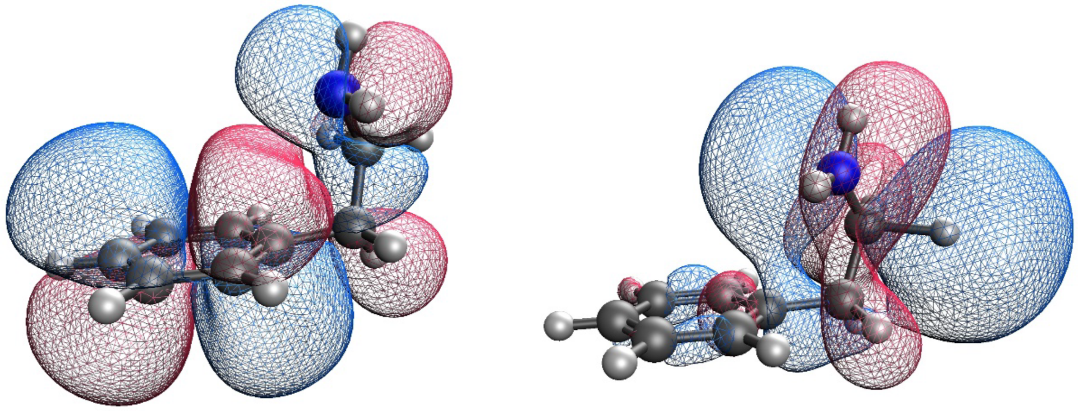

Figure S1. HOMO orbital of PEA at the stage of reactants (left) and at the transition state (right).

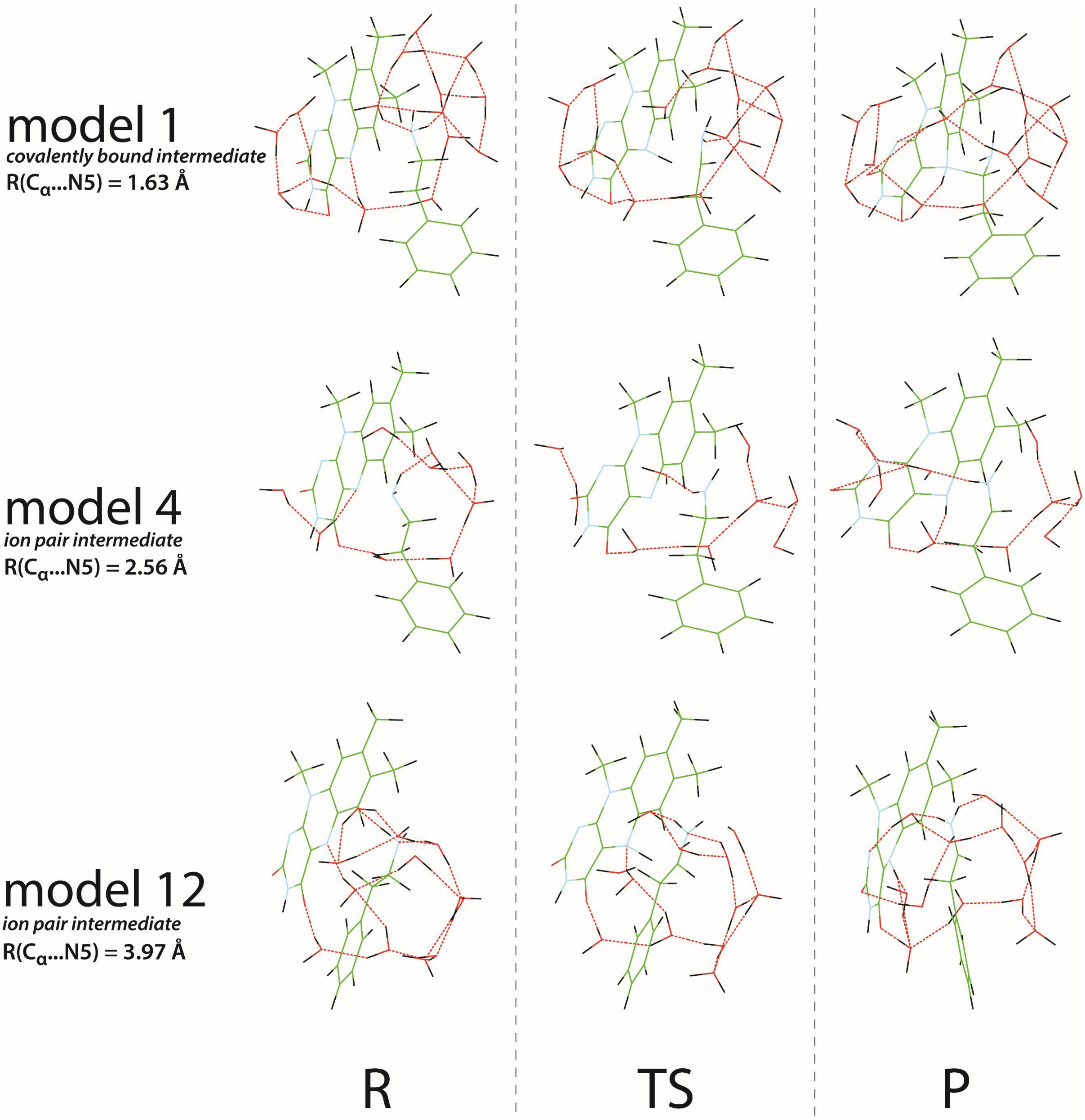

Figure S2. Characteristic structures – reactants (R), transition state (TS) and products (P) – of the hydride transfer step represented by three distinct models employing explicit solvation by water molecules. The model numbering is the same as in Table 1. Model 1 represents the case in which a covalently bound complex is formed as product. In models 4 and 12, dissociated (ion pair) PEA+…LFN- product is formed, with small (model 4) and large (model 12) C….N separation of ~2.6 and ~4.0 Å, respectively. Please note that the product (P) stage eventually corresponds to the reactive intermediate in the course of the reaction.

Table S1. Energy of the C_α—N5 bonding orbital, its occupancy, donor-acceptor interaction energy between the lone pair on the amino nitrogen and the C_α—N5 antibonding orbital, and the occupancy of the C_α—N5 antibonding orbital, computed by NBO analysis on various optimized structures of the covalently bound PEA...LFN complex, as function of the presence/absence of the surrounding water molecules (water ON/OFF). Pairs of values that do not support the generally observed trend of the C_α—N5 bond disrupting role of water molecules are marked in red.

structure no.	E (σ_{C-N}) [a. u.]		occ (σ_{C-N}) [e^-]		ΔE (LP(N) → σ^*_{C-N}) [kcal/mol]		occ (σ^*_{C-N}) [e^-]	
	water ON	water OFF	water ON	water OFF	water ON	water OFF	water ON	water OFF
**1**	-0.725	-0.744	1.967	1.970	36.97	34.32	0.167	0.166
**2**	-0.729	-0.750	1.968	1.971	35.69	33.16	0.162	0.160
**3**	-0.711	-0.735	1.968	1.971	33.09	31.15	0.161	0.165
**4**	-0.711	-0.735	1.968	1.971	33.12	31.18	0.161	0.165
**5**	-0.670	-0.712	1.962	1.967	47.92	41.60	0.205	0.191
**6**	-0.718	-0.739	1.966	1.970	37.15	34.81	0.169	0.168
**7**	-0.718	-0.739	1.966	1.970	37.15	34.82	0.169	0.168
**8**	-0.738	-0.758	1.971	1.974	29.60	28.84	0.146	0.152
**9**	-0.742	-0.770	1.972	1.975	27.32	27.21	0.137	0.144
**10**	-0.742	-0.770	1.972	1.975	27.31	27.20	0.137	0.144
**11**	-0.742	-0.765	1.972	1.974	28.17	27.98	0.141	0.148
**12**	-0.714	-0.738	1.967	1.971	34.91	34.73	0.173	0.167
**13**	-0.714	-0.738	1.967	1.971	34.92	34.76	0.173	0.167

Of the four tabulated parameters only occupancy of the antibonding orbital exhibits few exceptions that do not support this trend of weakening the C_α—N5 bond on interaction with surrounding water molecules. However, it should be noted that orbital occupancy (either of bonding or antibonding C_α—N5 orbital) appears to be much less sensitive to variations within the model than the bonding orbital energy or interaction energy between the amino nitrogen lone pair and the C_α—N5 antibonding orbital. Because all other parameters clearly support (without exception) a C_α—N5 bond weakening effect caused by the surrounding water molecules, we assume these exceptions have only little significance.

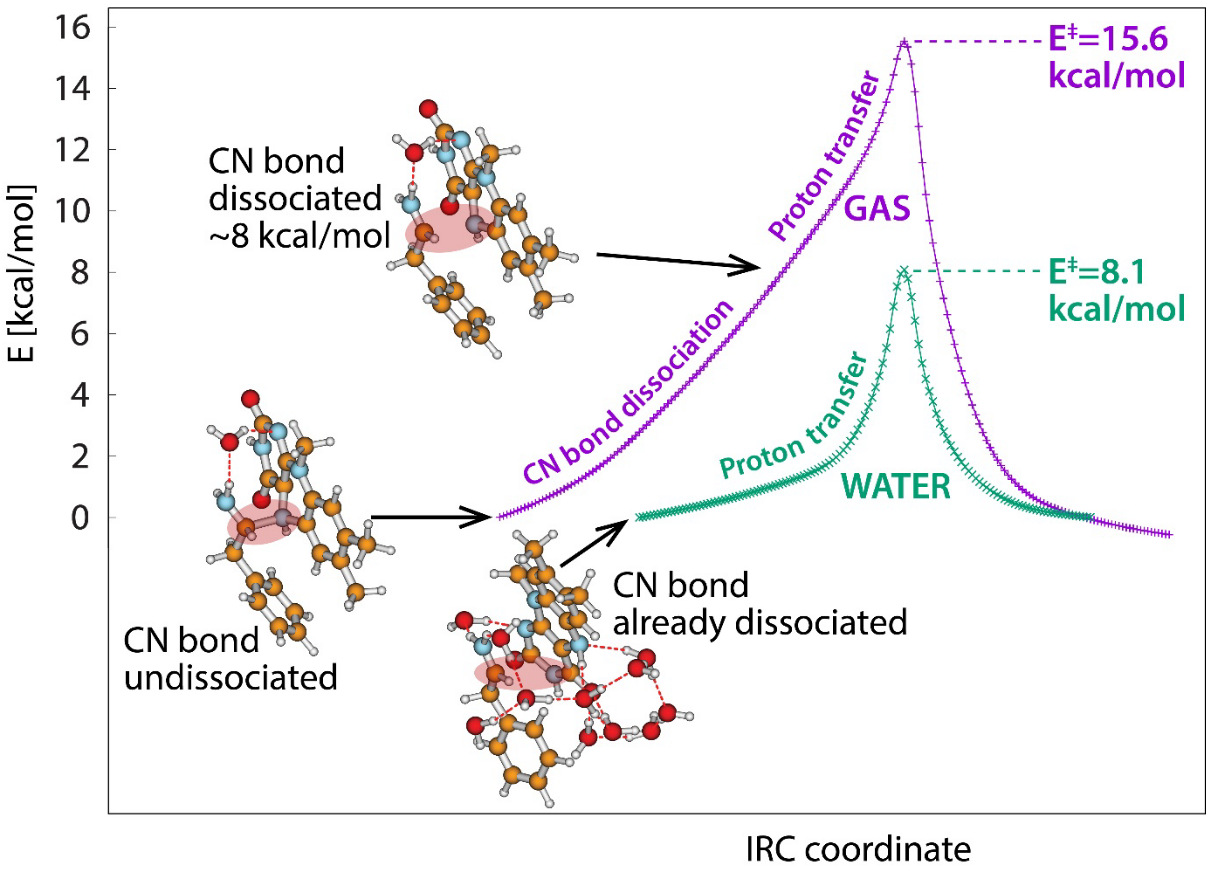

Figure S3. IRC profiles for the proton transfer step in the gas phase (purple line) and in the presence of explicit water molecules (green line). Characteristic structures are displayed, demonstrating that in the gas phase the reaction step starts with an undissociated C_α—N5 bond, requiring dissociation prior to proton transfer, a process costing ~8 kcal/mol. At the same time, in the presence of explicit water molecules, the C_α—N5 bond complex is already dissociated and the process starts directly with proton transfer, bearing no such cost and requiring substantially lower barrier.
